# BaGPipe: an automated, reproducible, and flexible pipeline for bacterial genome-wide association studies

**DOI:** 10.1186/s12866-026-04909-9

**Published:** 2026-03-27

**Authors:** Kuangyi Charles Wei, Beth Blane, Jacqueline Toussaint, Sandra Reuter, Michelle S. Toleman, Mili Estee Torok, Sharon J. Peacock, Ewan M. Harrison, Dinesh Aggarwal, William Roberts-Sengier

**Affiliations:** 1 Parasites and Microbes Programme, Wellcome Sanger Institute, Hinxton, UK; 2 Department of Genetics, University of Cambridge, Cambridge, UK; 3 Department of Medicine, University of Cambridge, Cambridge, UK; 4 European Bioinformatics Institute, Cambridge, UK; 5 Medical Center – University of Freiburg, Freiburg, Germany; 6 Cambridge University Hospitals NHS Foundation Trust, Cambridge, UK; 7 Department of Infectious Diseases, Imperial College London, London, UK

**Keywords:** Microbial GWAS, Nextflow pipeline, Pyseer, Whole genome sequencing, Genome-wide association study

## Abstract

**Background:**

Microbial genome-wide association studies (GWAS) are crucial for linking genetic variation to phenotypic traits in bacteria. However, current tools often involve complex manual processing, limited scalability, and fragmented workflows, which constrain large-scale or routine bacterial GWAS.

**Results:**

We developed BaGPipe, an automated and flexible bacterial GWAS pipeline built using Nextflow and incorporating Pyseer for association analysis. BaGPipe integrates pre-processing, statistical analysis, and downstream visualisation into a unified workflow that is reproducible and easy to deploy across diverse computational environments. BaGPipe was validated on a publicly available dataset of *Streptococcus pneumoniae* whole-genome sequences, and reproduced published findings with improved computational efficiency. BaGPipe was then applied to a dataset of *Staphylococcus aureus* whole-genome sequences, successfully identifying known and novel antibiotic resistance associations.

**Conclusions:**

By offering an accessible, efficient, and reproducible platform, BaGPipe accelerates bacterial GWAS and facilitates deeper exploration into the genetic underpinnings of phenotypic traits. BaGPipe is freely available at https://github.com/sanger-pathogens/BaGPipe.

**Supplementary Information:**

The online version contains supplementary material available at 10.1186/s12866-026-04909-9.

## Background

Genome-wide association studies (GWAS) have become an essential tool for uncovering the genetic basis of phenotypic traits across diverse organisms, particularly for investigating mechanisms of pathogenicity and resistance [1–5]. However, bacterial GWAS faces unique challenges due to horizontal gene transfer, complex population structures, and extensive genomic diversity, which complicate accurate genotype–phenotype associations [6–9]. Current bacterial GWAS tools often exacerbate these difficulties by requiring fragmented workflows, extensive manual intervention, and lacking scalability and reproducibility.

Executing bacterial GWAS requires analysing large genomic datasets to identify associations with phenotypes such as antimicrobial resistance (AMR), while accounting for factors like population structure and statistical power [10]. Current bacterial GWAS tools fall into three categories [9, 11, 12]: phylogenetic, non-phylogenetic, and machine learning approaches (See Supplementary Table 1). Phylogenetic tools like Scoary [13] and TreeWAS [14] rely on well-defined phylogenetic structures and are suitable for datasets where recombination can be mitigated but are less practical for highly diverse species or large datasets. Non-phylogenetic tools such as Bugwas [15] and SEER [16] adapt concepts from human GWAS to develop phylogeny-independent methods. While computationally efficient and scalable, they face challenges like elevated false-positive rates due to insufficient correction for population structure. Machine learning GWASs tools are rarely used, and no benchmarking has been performed.

Among non-phylogenetic tools, Pyseer [17] effectively addresses population structure and mitigates false positives by incorporating a linear mixed model [18]. By leveraging dimensionality reduction techniques like multidimensional scaling (MDS) and employing k-mer based association studies, Pyseer has emerged as a standard tool in the field due to its robustness and speed, especially in identifying genetic variants with small effect sizes [12, 19–22].

**Fig. 1:**
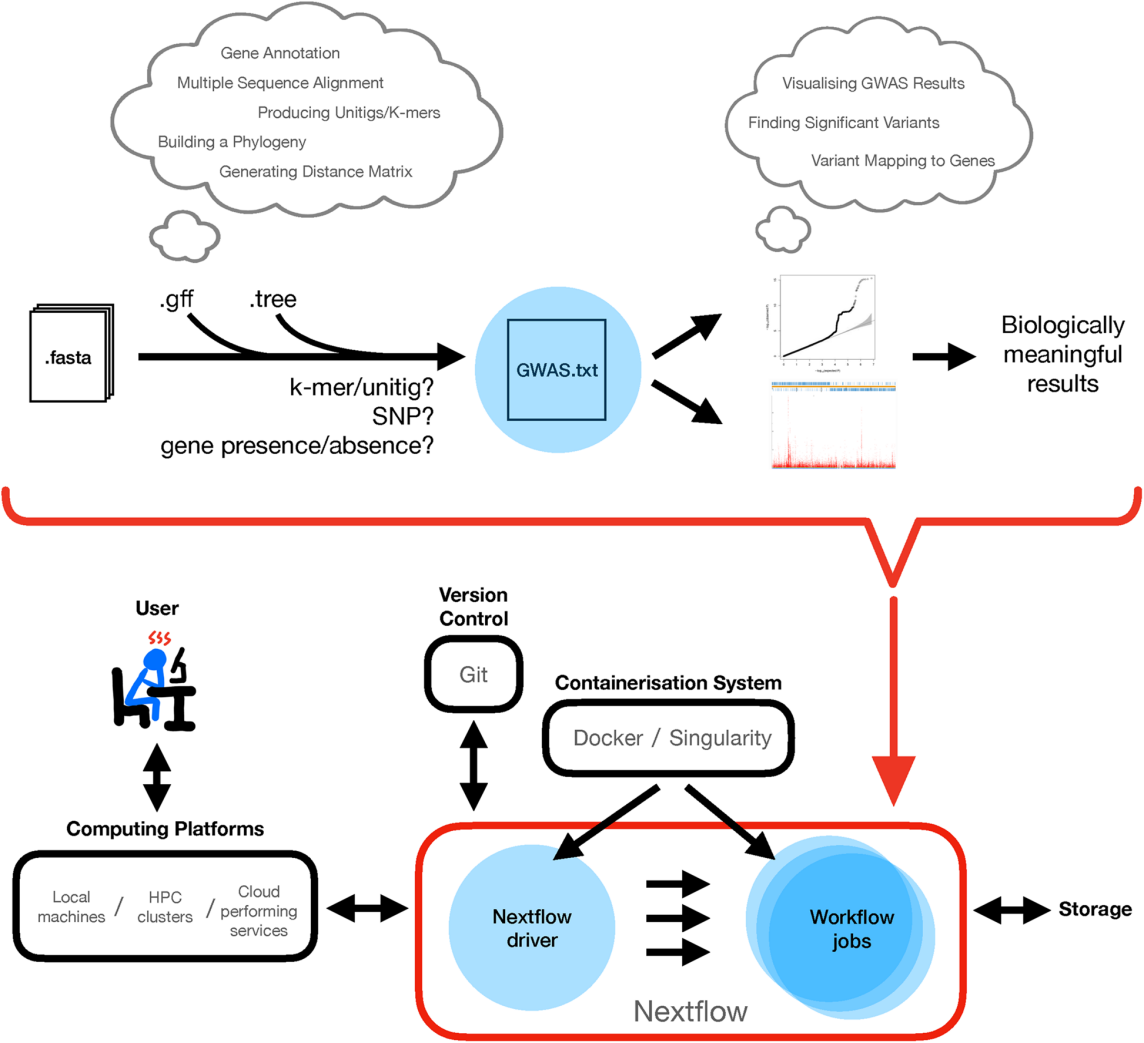
Schematic overview of the problem addressed and the solution by BaGPipe. Top: A comprehensive bacterial GWAS workflow involves miscellaneous pre-processing steps and various post-processing analyses, yet most bacterial GWAS tools only achieve the association study (shown by a blue circle). Bottom: The objective is to compile all relevant tools into a Nextflow pipeline, which can be easily deployed by users across computing platforms. The quantile–quantile plot (Q-Q plot) and the Manhattan plot are taken from the Pyseer tutorial [30]

Despite these advancements, executing bacterial GWAS analyses remains hindered by significant technical challenges. Pyseer and similar tools [23–25] lack integrated solutions for crucial pre-processing steps –– such as genome annotation, phylogenetic inference, or distance matrix generation –– and downstream analyses like result visualisation and annotation (Fig. 1). These steps demand expertise across multiple bioinformatics tools, each with its own dependencies and idiosyncrasies, leading to an increased risk of error, wasted computational resources, and ultimately, a diversion from biological interpretation towards technical troubleshooting. Existing pipelines, such as bacterialGWAS [26], DBGWAS [23] and GEMMA kmer_pipeline [27], attempt to address specific aspects of bacterial GWAS but remain limited by their narrow scope, fragmented workflows, or lack of scalability. A recent Snakemake pipeline, microGWAS [28], offers a more comprehensive wrapper around Pyseer, but it does not fully automate pre- and post-processing steps or offer the modular scalability enabled by the popular modern workflow manager, Nextflow [29].

To address these unmet needs, we present BaGPipe, a highly scalable and reproducible pipeline built on Nextflow [29] (Fig. 1). BaGPipe fully leverages Pyseer for bacterial GWAS analysis while encompassing the entire GWAS workflow. We validate BaGPipe by replicating previously published findings in a well-characterised *Streptococcus pneumoniae* dataset [30–32] and further examine its utility by exploring novel genetic associations in a clinically relevant *Staphylococcus aureus* genomic dataset [33]. We then benchmark BaGPipe with AURORA [34], a Machine Learning tool, and microGWAS [28], a Snakemake pipeline, on the same *S. pneumoniae* dataset to compare their outputs and computational efficiencies.

## Methods

### Pipeline overview

BaGPipe is a bacterial GWAS pipeline designed to integrate a wide array of bioinformatics tools into a unified workflow that can efficiently handle pre-processing steps to post-processing analysis and visualisation. The pipeline was developed to address computational challenges and facilitate genome-wide association studies on bacterial datasets in a reproducible and user-friendly manner. The pipeline utilises Nextflow [29] for workflow management, ensuring compatibility across diverse computational environments while optimising resource usage. A step-by-step tutorial and comprehensive documentation are available online (https://github.com/sanger-pathogens/BaGPipe).

**Fig. 2:**
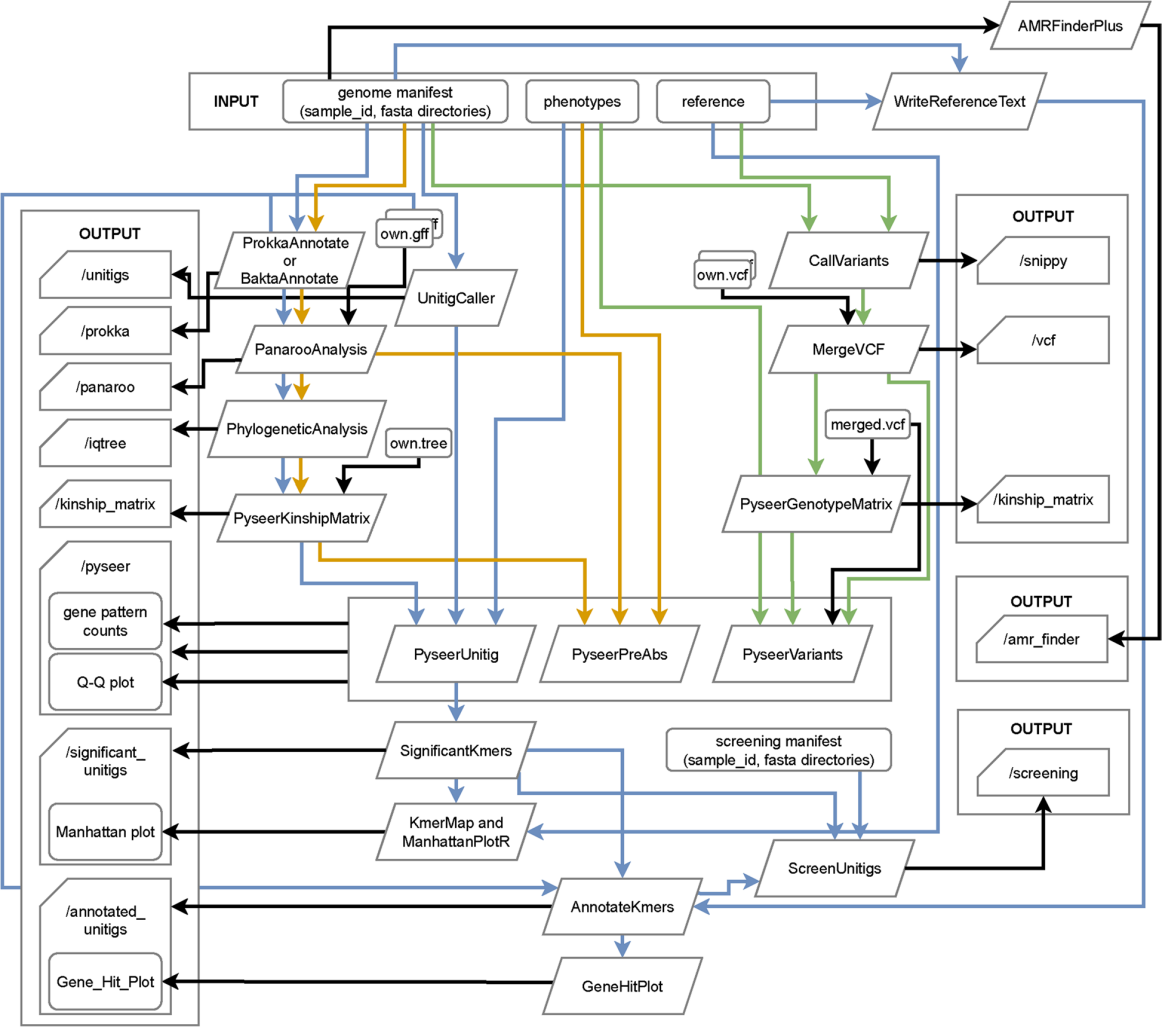
Overview of the BaGPipe pipeline. Input files are displayed on the top and output files on the sides. Alternative input files (GFF file, phylogenetic tree file, or VCF file) can be provided using appropriate option parameters. In a comprehensive analysis using the unitig mode, there will be eight output folders. The colour defines the genotype mode of analysis (blue: unitig/k-mer; yellow: gene presence and absence; green: short variations, including SNPs and Indels)

BaGPipe supports different entry points to provide maximum flexibility for users (Fig. 2), allowing for the integration of their own input data, such as phylogenetic trees or variant call formats (VCFs). This feature allows BaGPipe to accommodate diverse user requirements, reducing the burden of having to repeat computationally intensive pre-processing tasks.

### Pipeline implementation and reproducibility

To ensure ease of use and reproducibility, BaGPipe was developed using Docker and Nextflow [29]. Docker standardises the software environment, encapsulating all essential tools and specific versions into a single container for consistency across platforms. Nextflow manages the modular pipeline, allowing users to execute the workflow with a single command while specifying input files and analysis settings:

- # install pipeline
- nextflow pull sanger-pathogens/bagpipe
- # run pipeline
- nextflow run sanger-pathogens/bagpipe --help

For high-performance computing (HPC) environments, BaGPipe supports the Load Sharing Facility (LSF) executor, enabling efficient parallel processing by dispatching each task as an independent job. It dynamically allocates memory and CPU based on task needs and can escalate resource requests if a job fails due to underestimation.

### User accessibility and configuration

The pipeline includes a default configuration file with all the key options and parameters necessary for running BaGPipe. This configuration is easily modifiable: non-experienced users only need to adjust key parameters, while advanced users can modify any parameter by editing the source code or profile configuration. As BaGPipe is implemented in Nextflow, it can be deployed on a wide range of HPC infrastructures and schedulers, including LSF and SLURM, by supplying an appropriate institutional profile. This design ensures that BaGPipe remains both accessible and highly adaptable to diverse compute environments and research workflows.

### Pre-processing

In its most comprehensive mode, BaGPipe starts with genome assemblies and automates pre-processing, including the generation of k-mers or unitigs, genome annotation, pangenome analysis, and phylogenetic analysis. This pre-processing stage is supported by Prokka (v.1.14.6) [35] and Bakta (v.1.11.3) [36] for annotation, Panaroo (v.1.5.1) [37] for constructing pangenomes and creating a multiple sequence alignment of core genes, and IQ-TREE (v.2.2.6) [38] for generating a core genome phylogeny. The output from these steps provides the essential elements for subsequent analysis, such as annotated assemblies, a core phylogenetic tree, and a distance matrix that reflects the genetic relationships within the dataset.

### Association analysis

BaGPipe employs Pyseer (v.1.3.11) [17], a Python implementation of SEER [16], to conduct the association study, leveraging a linear mixed model to identify genotype–phenotype relationships. The typical bacterial GWAS requires three core types of inputs: genotypes, phenotypes, and an interaction or population structure correction. In BaGPipe, users can conduct association analyses using different genotyping approaches, including unitigs, k-mers, SNPs and indels, which provide flexibility in how genomic variation is represented.

The linear mixed model in Pyseer controls for population structure using kinship matrices derived from phylogenetic analysis. Alternatively, a pairwise distance matrix can be generated from genome assemblies using Mash [39], which is then employed to manage population stratification. BaGPipe supports Pyseer’s k-mer/unitig-based association study, which is recognised as a best practice for bacterial GWAS due to its capacity to handle high genetic diversity while controlling for population structure [30]. This k-mer/unitig approach aids in controlling population structure by providing a comprehensive genetic representation of each isolate, capturing all types of genetic variations –– including SNPs, indels, and structural variants. By using k-mers/unitigs to quantify genetic similarities among isolates, a more accurate kinship matrix can be constructed, reflecting the true genetic relationships based on the presence or absence of k-mers across genomes.

Q-Q plots are generated to visualise the *p*-value distribution against the null hypothesis, ensuring that significant associations are not artefacts of confounding factors, such as population structure, phylogenetic relatedness, horizontal gene transfer, or technical biases. Additionally, as a good practice inherited from using Pyseer, BaGPipe provides a count of genotype patterns to guide users on whether corrections for multiple testing are necessary, if k-mers or unitigs are used as the genotyping approach.

### Post-processing and visualisation

BaGPipe facilitates intuitive analysis by automatically processing significant unitigs or other genomic elements identified during the association analysis. It generates Manhattan plots to visualise the genomic locations of significant associations and annotates these markers using both reference and draft assemblies. The output includes a "gene_hits.tsv" file summarising significant unitigs and their gene annotations, providing insights into potential biological functions.

BaGPipe enriches the final output by annotating genes in the neighbourhood of significant markers, which aids in biological interpretation. Additionally, a default R script (adapted from the documentation of Pyseer [30]) is provided to allow users to create custom visualisations of the data, further simplifying downstream exploration and interpretation.

### Screening of GWAS-identified loci on external datasets

BaGPipe includes an optional module for screening GWAS-identified k-mers or unitigs in independent external cohorts. The module accepts as input the list of statistically significant loci identified during the discovery GWAS and a user-provided manifest of genome assemblies from the screening dataset. Each of these genomes is screened for the presence or absence of these loci using a unitig-based matching approach. The module produces summary outputs including per-sample counts of detected loci, per-locus prevalence across the screening cohort, and optional gene-level summaries when annotations from the discovery phase are available. This functionality enables screening of further genomes for potential virulence or resistance traits without additional manual processing.

### Optional annotation of antimicrobial resistance genes

To allow for comparison with known antimicrobial resistance (AMR) genes and allow users to explore novelty within their analyses, BaGPipe provides an optional functional annotation module that integrates AMRFinderPlus for the identification of known AMR genes from genome assemblies. When enabled, AMRFinderPlus (v.4.0.23) [40] is run automatically on all samples, generating per-sample AMR profiles and a cohort-level summary of detected resistance determinants.

### Implementation of BaGPipe on the *Streptococcus pneumoniae* dataset

To assess BaGPipe’s ability to identify resistance-associated variants, we applied it to a well-characterised *S. pneumoniae* dataset (*n* = 616) on beta-lactam resistance. This dataset was chosen as a benchmark due to its prior use in bacterial GWAS [30–32], allowing for comparisons with existing methods like Pyseer. Different to the Pyseer tutorial [30], BaGPipe was run with unitigs (maximal non-branching paths in a compacted de Bruijn graph) instead of k-mers to reduce sequence redundancy while maintaining the integrity of the analysis. For inputting to BaGPipe, we made the required genome manifest CSV file indicating the paths to all assemblies. To conduct significant unitig analysis, we also made a reference manifest CSV file. BaGPipe was executed with the LSF executor.

### Implementation of BaGPipe on the *Staphylococcus aureus* dataset

To evaluate BaGPipe’s performance in a different genomic context, we applied it to a methicillin-resistant *S. aureus* (MRSA) dataset [33] (metadata see Supplementary Table 2), aiming to identify genetic determinants of antibiotic resistance in a real-world genomic surveillance setting. To quality-control the assemblies, we ran the FASTQ files (*n* = 520) through bacQC (v.1.2, https://github.com/avantonder/bacQC), which trimmed sequences using fastp [41]. Inspection on the output QC file confirms a slight improvement in the quality of the data, with fewer adapter sequences and PCR duplicate artifacts. From the Kraken2 [42] and Bracken [43] reports, two obviously contaminated samples were identified and excluded from the data for further analysis (see Supplementary Table 3). Next, we forwarded the FASTQ files (*n* = 518) to assembleBAC (v.1.2, https://github.com/avantonder/assembleBAC). It was observed that the smallest N50 is 72.2 Kbp and all 518 assemblies have length 2.8–3.0 Mbp. We discarded samples with less than 90% reads matching to *S. aureus* and those with < 30 × coverage from onward analyses, as well as removing assemblies with an N50 value < 10 Kbp, length of less than 2.6 Mbp or greater than 3.0 Mbp, or with a spuriously high number of contigs. No other samples were excluded based on these criteria (518 samples retained).

We prepared the appropriate manifest files and other input files and executed BaGPipe using the LSF executor. Analyses were conducted to identify the genetic basis of resistance to eight antibiotics: oxacillin, ciprofloxacin, erythromycin, fusidic acid, clindamycin, tetracycline, gentamicin, and mupirocin. Antibiotic resistance in the *S. aureus* dataset was recorded as binary. Details of isolate collection, whole-genome sequencing and antimicrobial susceptibility testing are available in the published study [33].

To compare our results, we ran AMRFinderPlus (v.3.11.18) [40] for prediction of antimicrobial resistance genes from known databases. For each antibiotic, we compiled AMRFinder results and extracted significant gene-level associations at FDR ≤ 0.05 from BaGPipe runs. Gene symbols were harmonised by lower‑casing, removing parentheses, stripping variant suffixes (e.g., *parC_S80F* changed to *parC*). We computed a set of agreement metrics between BaGPipe significant genes and AMRFinder catalogue genes, including precision (fraction of BaGPipe genes that are in the catalogue), recall (fraction of catalogue genes recovered by BaGPipe), Jaccard (intersection over union), and F1 score (harmonic mean of the precision and recall). AMRFinder per‑gene counts across the 518 isolates were used to estimate prevalence (= count/518). BaGPipe’s avg_beta was used as the effect size proxy (log‑odds).

### Benchmarking BaGPipe against other GWAS tools on the on the *Streptococcus pneumoniae* dataset

To benchmark BaGPipe against a machine learning approach, we applied AURORA (v.1.0.0) [34] to the *S. pneumoniae* dataset. Pangenome presence/absence was generated with Panaroo (v.1.5.1) [37] in Roary-compatible mode and the resulting gene_presence_absence_roary.csv was used as the feature matrix for both AURORA and cross-method mapping. For gene-level benchmarking, AURORA features (group_####) were mapped to gene symbols using the Roary “Non-unique Gene name” and “Annotation” fields; when multiple aliases were present, all were retained and the maximum score per gene was used. Features that could not be mapped beyond “group_####” were excluded to ensure comparability with BaGPipe gene identifiers. A maximum likelihood phylogeny was inferred from the core genome alignment and pruned to genomes with phenotype labels prior to AURORA analysis. AURORA’s phenotype module was used to identify non-typical or mislabelled strains via a bagged random forest with outlier detection, with per-bag proximities and summary plots inspected to assess class balance and outliers. GWAS was performed using the Panaroo Roary-format matrix (type_bin_mat = "panaroo") and the pruned phylogeny, yielding per-feature performance against the resistant class. Gene-level AURORA evidence was summarised by the maximum F1 score per gene (primary metric), with additional metrics (precision, recall, standardised residuals) retained for sensitivity analyses and figure annotation. Cross-method comparison used AURORA F1 scores and BaGPipe gene hits as ranking metrics, with overlap assessed by set intersection, Jaccard distance, and Spearman rank correlation within the overlap.

To benchmark BaGPipe against a Snakemake-based GWAS pipeline, we ran microGWAS (v.0.6.2) [28] on the same *S. pneumoniae* dataset using Bakta (v.1.11.3) [36] generated GFF annotations produced from BaGPipe’s annotation step. microGWAS was executed end-to-end with its unitig mode, producing per-feature and gene-level outputs. To compare gene hits, unitig‑level microGWAS results were mapped to genes via a Panaroo (Roary‑format) dictionary with stringent tokenisation, excluding “bad-chisq” rows and unmapped “group_####” labels, scoring genes by the maximum − log_10_(*p*-value), ranking both methods by descending score, and quantifying agreement via intersection size, Jaccard index, and Spearman rank correlation on the overlap. As both pipelines were run on an HPC, Slurm accounting and workflow logs were collected for all runs on the same HPC profile to record wall‑time, peak memory and disk usage. Furthermore, BaGPipe was run on one published *Escherichia coli* virulence dataset (*n* = 370) [44] originally reported by the microGWAS authors [28] and the results were compared.

### Statistical comparison of gene sets and rankings

Set overlap was quantified using the Jaccard index, defined as the number of shared genes divided by the number of genes present in either set. For comparisons with the curated AMR catalogue, precision was the proportion of BaGPipe genes found in the catalogue, recall was the proportion of catalogue genes recovered by BaGPipe, and the F1 score was the harmonic mean of precision and recall. For antibiotic-specific analyses, precision, recall, and F1 were computed per antibiotic; precision and recall were summarised across antibiotics using the median and range, and macro-averaged F1 was summarised as the mean and range of the per-antibiotic F1 values. If a denominator was zero, the metric was treated as missing and excluded from summaries; if a numerator was zero, the metric was set to zero. Ranking concordance was assessed in two ways. First, top-list overlap was computed by comparing the number of shared genes between the two top lists for sizes from 10 to 100 in steps of 10; for comparability across sizes, a top-list Jaccard was also reported using the same definition of shared over either list. Ties at the boundary were included and truncated deterministically with a fixed seed to ensure reproducibility. Second, Spearman rank correlation was computed on the overlapping genes only, using average ranks for ties and a two-sided P value for the null of no monotonic association. Where significance of set overlap was reported, it was evaluated with a two-sided Fisher exact test on a two-by-two table of set membership, with false discovery rate control by the Benjamini–Hochberg procedure. All analyses were performed in R, with two-sided tests and fixed random seeds for any tie-breaking.

## Results

### Validation of BaGPipe by reproducing analyses from Pyseer

We validated BaGPipe on 616 *S. pneumoniae* isolates, the dataset used in the Pyseer tutorial [30]. This dataset has previously been used to identify genes associated with penicillin resistance, providing a benchmark for bacterial GWAS. By analysing the identical isolates, we aimed to assess BaGPipe’s ability to replicate these findings and evaluate its performance against established methods.

**Fig. 3:**
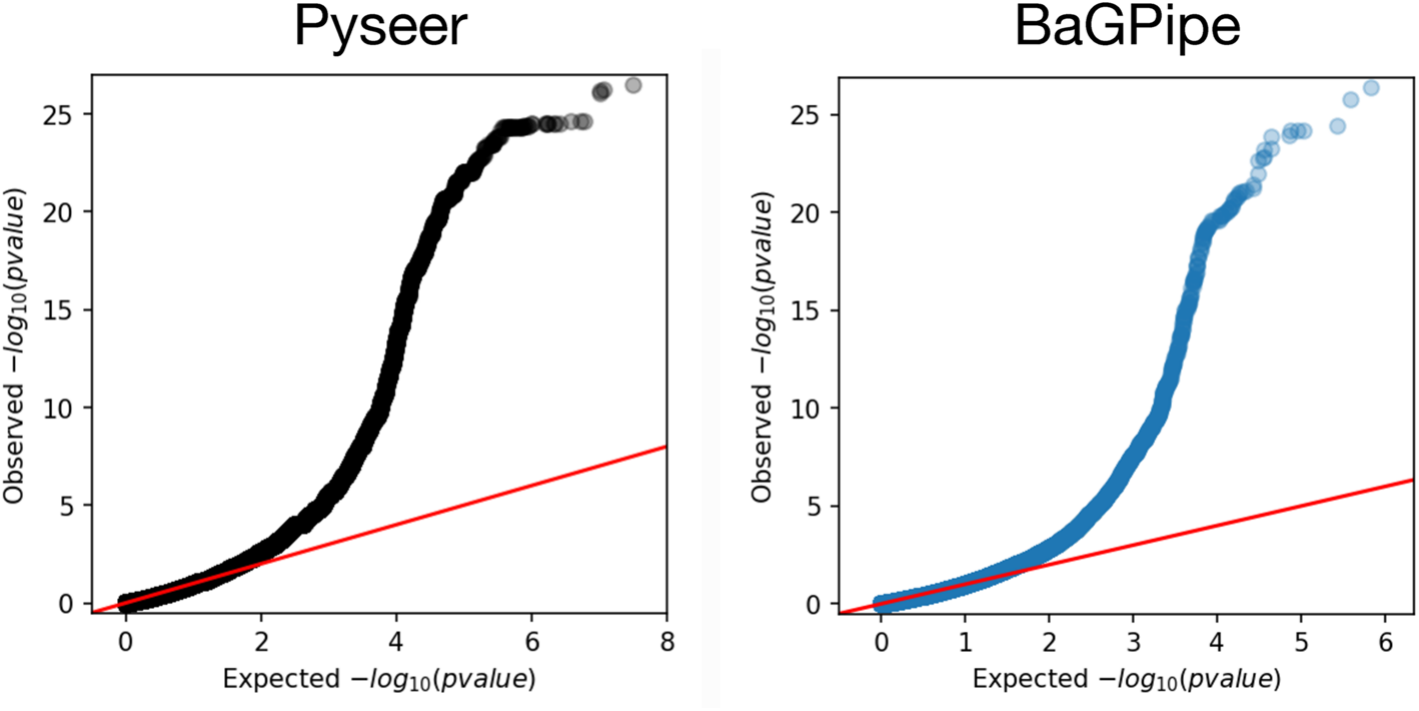
Comparison of the Q-Q plots from BaGPipe and from Pyseer on the *Streptococcus pneumoniae* dataset. The Q-Q plots show that observed − log_10_(*p*-values) are not inflated at low − log_10_(*p*-values) and there is an absence of any poorly controlled confounding population structure (they would appear as big “steps” deviating from the diagonal line). The position of the points being above the null hypothesis (the diagonal line) indicates significant k-mers/unitigs associated with penicillin resistance. The Q-Q plot produced from Pyseer was sourced from the Pyseer tutorial [30]

BaGPipe successfully identified 337,885 unique unitig patterns from a total of 740,945 unitigs tested. Recognising that many unitigs are highly correlated due to sequence overlap, we determined a significance threshold of 1.48E-07 using a Bonferroni correction based on the number of unique unitig patterns (0.05/337,885). This approach aligns with the Pyseer tutorial, which suggests adjusting the significance threshold based on the effective number of independent tests. We then applied this threshold to filter significant unitigs –– those with *p*-values below 1.48E-07 were considered significant. By using this adjusted threshold, we effectively controlled for multiple testing without being overly conservative, ensuring that the identified associations are statistically robust. The Q-Q plot generated by BaGPipe closely resembled that produced in the Pyseer tutorial (Fig. 3), demonstrating consistency in managing population structure and ensuring the absence of poorly controlled confounders.

**Fig. 4:**
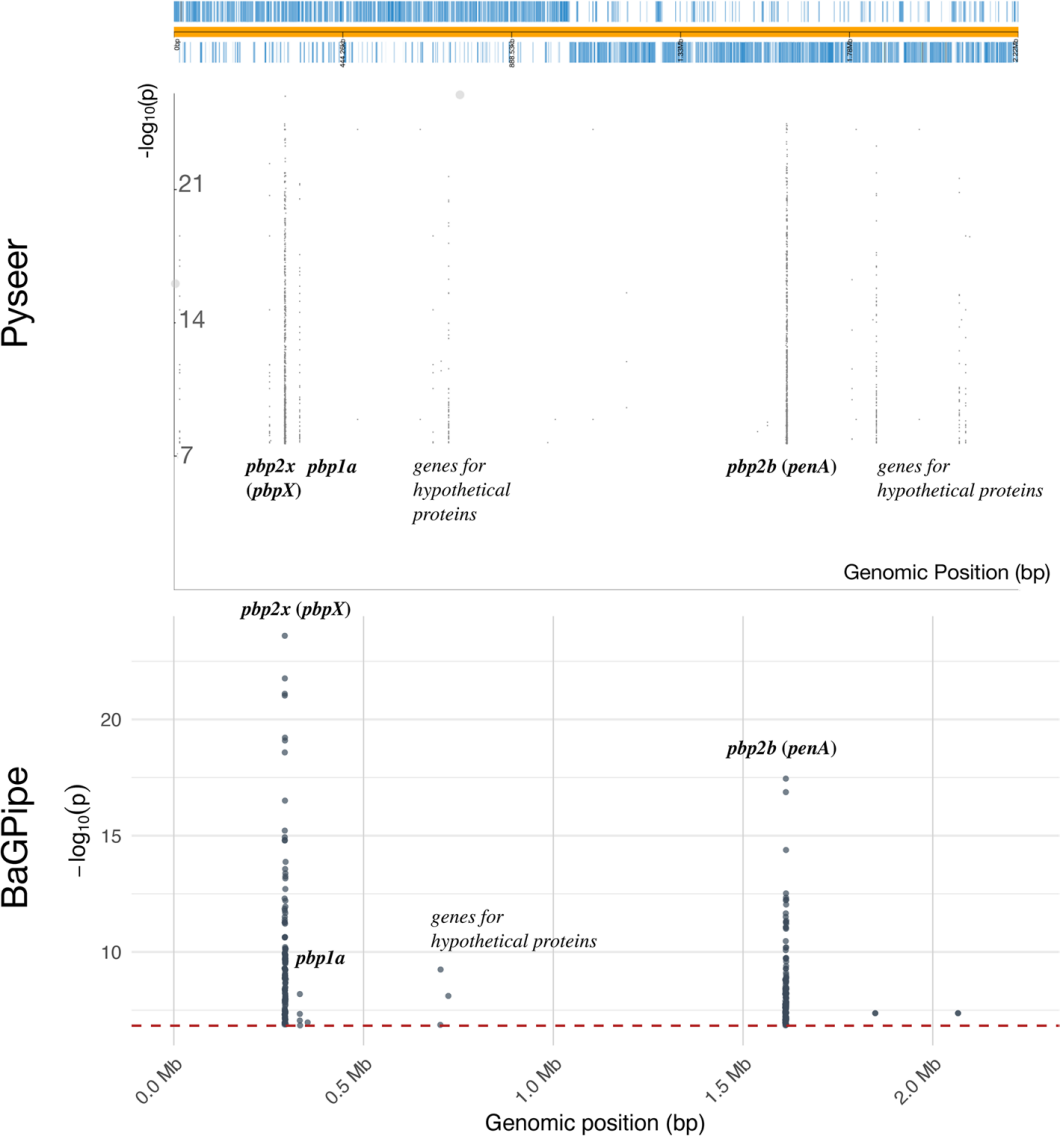
Comparison of the Manhattan plots from BaGPipe and from Pyseer on the *Streptococcus pneumoniae* dataset. The plot is again consistent with that in the Pyseer tutorial, showing two peaks, corresponding to the two *pbp* genes. The strongest peak represents the locus coding for the putative penicillin binding protein 2x (*pbp2x*); the second strongest peak represents *pbp2b* gene. The Manhattan plot produced from Pyseer, visualised via Phandango [45], was sourced from the Pyseer tutorial [30]

The subsequent significant unitig analysis conducted by BaGPipe identified 573 significant unitigs, in contrast to the 5,327 significant k-mers reported in the Pyseer tutorial. This notable reduction demonstrates the effectiveness of using unitigs to reduce sequence redundancy while retaining significant biological information. The significant unitigs were mapped to the reference genome, and a Manhattan plot was generated, showing two distinct peaks corresponding to the two penicillin-binding protein (pbp) genes: *pbp2x* and *pbp2b* (Fig. 4). These are the hits found in the previous studies and they are known genes involved in penicillin resistance [30–32].

Not all significant unitigs mapped to a single reference, underscoring the need for multiple high-quality reference genomes to ensure comprehensive annotation and reliable downstream analyses. The Manhattan plot generated by BaGPipe was consistent with that from the Pyseer tutorial, although certain peaks observed in the k-mer analysis were attenuated in the unitig analysis. Because unitigs compact overlapping k-mers, the effective number of independent tests and the influence of rare features differ between representations; in this dataset, this primarily narrows signals to *pbp2x*/*pbp2b*, while neighbouring *recR*/*ddl* signals appear linkage disequilibrium (LD)-consistent and parameter-sensitive.

**Fig. 5:**
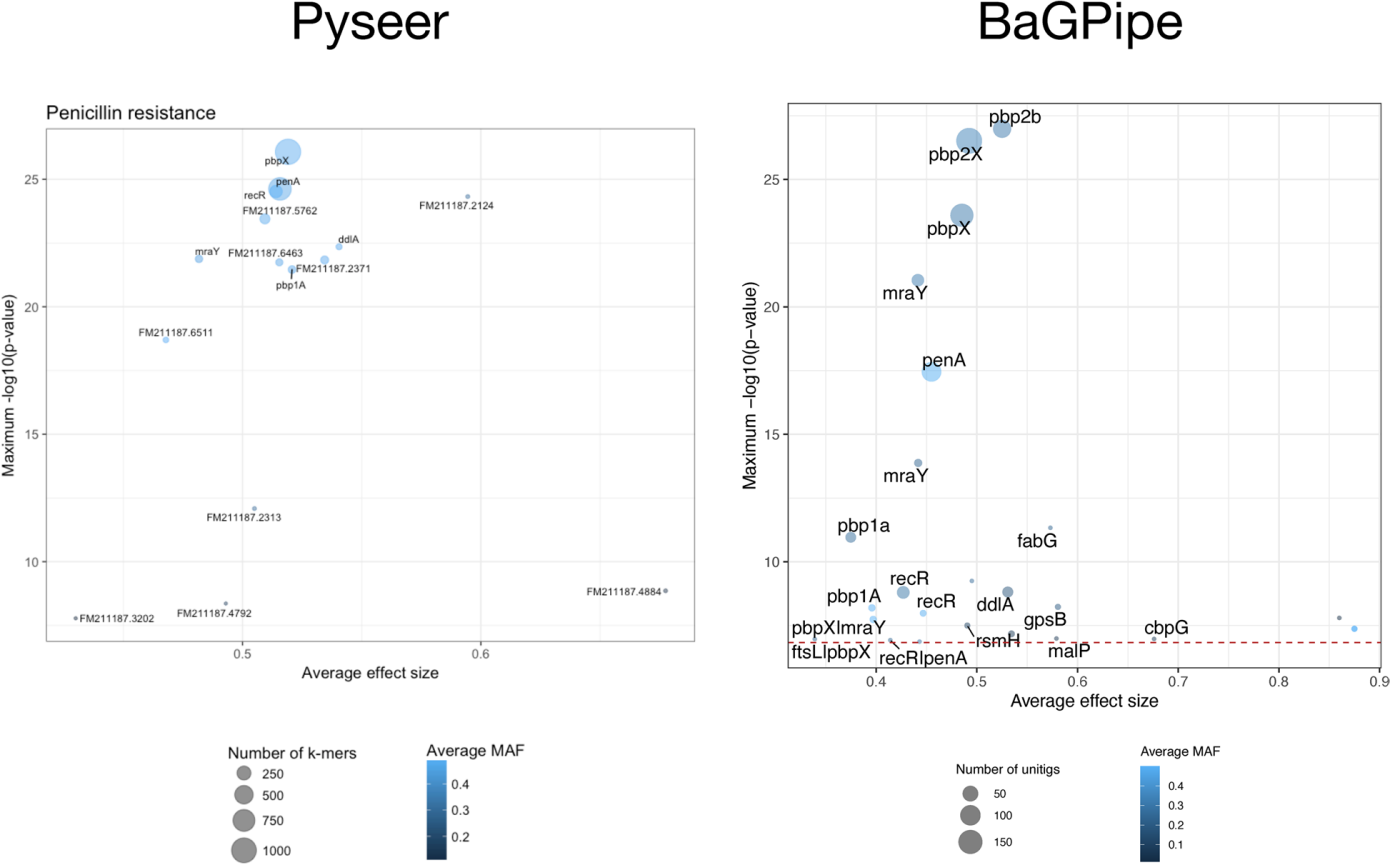
Comparison of the gene-hit plots from BaGPipe and from Pyseer on the *S. pneumoniae* dataset. The x-axis represents the average effect size, while the y-axis shows the maximum − log_10_(*p*-value), highlighting the statistical significance of gene associations. The size of the circular dots indicates the number of k-mers/unitigs involved in each association, providing insight into the genomic support for each hit. The colour scheme, ranging from lighter to darker shades of blue, illustrates the average minimum allele frequency (MAF), with darker shades indicating a lower MAF. BaGPipe accurately replicated the findings from Pyseer, confirming *penA* (*pbp2b*) and *pbpX* (*pbp2x*) as the most significant gene hits. The gene-hit plot produced from Pyseer was sourced from the Pyseer tutorial [30]

After annotating the significant unitigs, BaGPipe produced a list of "gene hits," which included unitigs that either resided within or were adjacent to specific coding regions. The gene-hit plot summarising these results closely matched that of the Pyseer tutorial (Fig. 5). Key hits such as *penA* (*pbp2b*) and *pbpX* (*pbp2x*) were consistently identified across both analyses, demonstrating BaGPipe's reliability. Interestingly, the *recR* and *ddl* gene, identified as significant in the Pyseer analysis, showed a lower significance in the BaGPipe-derived results. *recR* encodes a protein involved in DNA repair, specifically the RecFOR pathway for homologous recombination [46], which may occasionally show spurious associations due to proximity to key resistance genes. This discrepancy between using k-mers and unitigs suggests that differences at *recR* reflect LD-driven, representation-dependent associations: prior work shows the *pbp2b*-*recR*-*ddl* block (*ddl* lies ~ 0.8 kb from *pbp2b* with *recR* between them) frequently recombines under β-lactam selection, so signals at *recR* can arise by hitchhiking with *pbp2b* [47]. Under a unitig representation and pattern-based thresholds these neighbouring signals are attenuated, while the *pbp2x*/*pbp2b* peaks remain.

Reproducing this analysis from raw reads, BaGPipe used approximately 191 h of total CPU time, with a peak remote-access memory (RAM) usage of 3.5 GB, a mean RAM usage of 574 MB, and an overall run time of about 23 h and 11 min. When excluding steps such as annotation, pangenome analysis, and phylogenetic analysis –– assuming these are provided as input –– the CPU time would be reduced to approximately 19 h.

### Implementation of BaGPipe on a *S. aureus* dataset

**Fig. 6:**
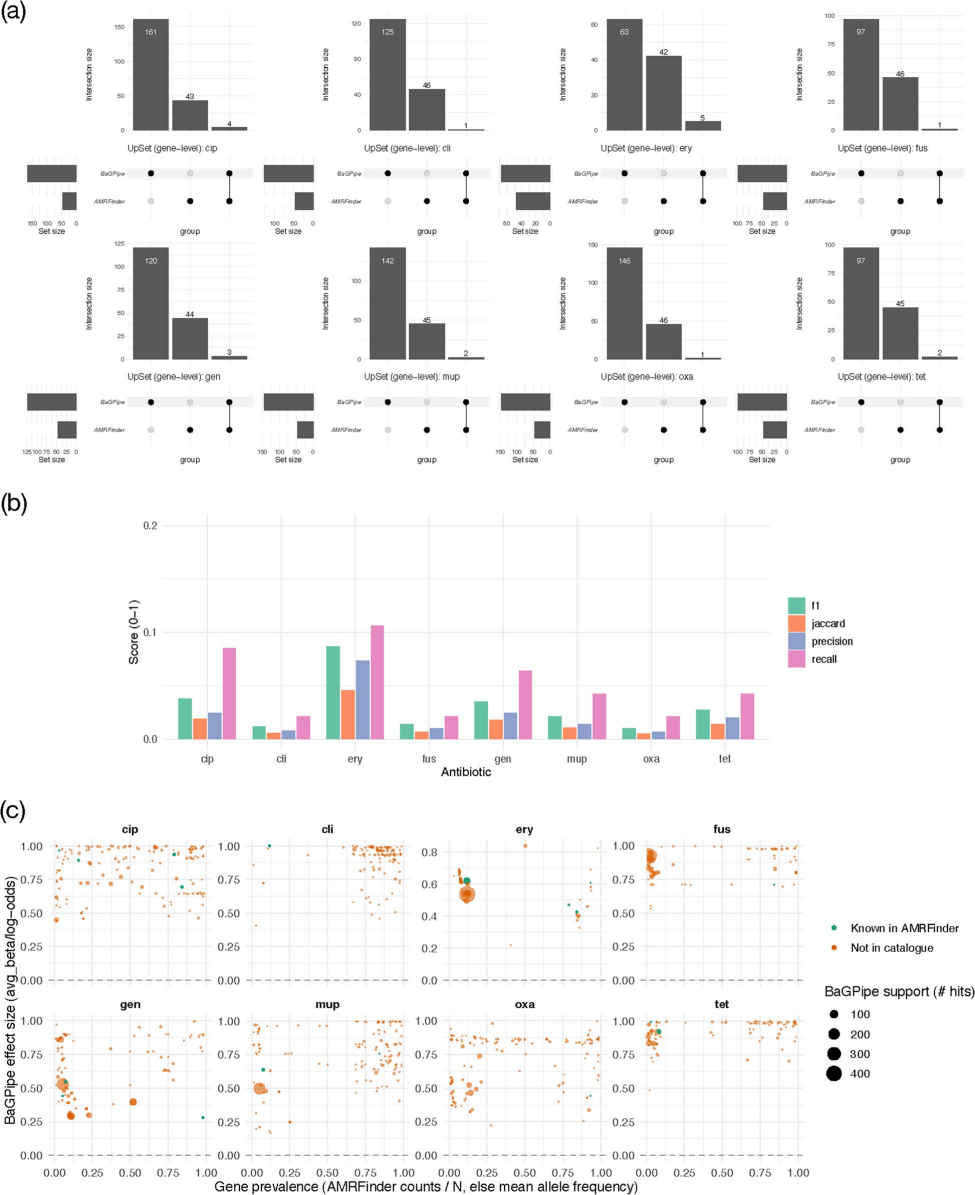
Agreement between BaGPipe GWAS hits and AMRFinderPlus across eight antibiotics in *S. aureus*. **a** UpSet plots display, for each antibiotic, the gene-level intersection and exclusive sets: BaGPipe-only (significant GWAS hits, FDR ≤ 0.05), AMRFinder-only (catalogue genes), and the intersection. Gene symbols were harmonised as described in Methods. **b** Overlap metrics per antibiotic (Jaccard index, precision, recall, F1) computed on the gene sets in (**a**). **c** BaGPipe effect size (avg_beta/log-odds) versus gene prevalence (AMRFinder counts/518 isolates), coloured by AMRFinder status; filled symbols denote FDR ≤ 0.05. AMRFinder counts provide prevalence for catalogue genes; prevalence is derived from mean allele frequency across tests contributing to the gene for BaGPipe-only genes. Point size encodes BaGPipe support (gene hits). cip, ciprofloxacin; cli, clindamycin; ery, erythromycin; fus, fusidic acid; gen, gentamicin; mup, mupirocin; oxa, oxacillin; tet, tetracycline

BaGPipe was further evaluated using high-quality sequences from 518 *Staphylococcus aureus* samples tested against seven antibiotics [33]. It successfully identified significant genetic associations with resistance phenotypes for multiple antibiotics. To cross-validate these findings, AMRFinderPlus [40] was employed to identify known AMR genes. BaGPipe's results were compared with AMRFinderPlus predictions, confirming the presence of established AMR genes, such as *gyrA*, *parC*, and *parE* for ciprofloxacin, and *ermA* for erythromycin (Fig. 6a). Set overlap was modest (Fig. 6b), with a median Jaccard of 0.012 (range 0.005–0.045) reflecting the disparity in set sizes and the broader scope of GWAS compared with a curated catalogue. Precision (fraction of BaGPipe genes present in the catalogue) had a median of 0.017 (range 0.0068–0.0735), whereas recall (fraction of catalogue genes recovered by BaGPipe) had a median of 0.0426 (range 0.021–0.106). The resulting macro-F1 averaged 0.030 across antibiotics (range 0.010–0.087). Intersections comprised 1–5 genes per antibiotic (e.g., ciprofloxacin = 4; erythromycin = 5), consistent with recovery of canonical determinants alongside additional BaGPipe-only candidates, suggesting novel resistance mechanisms that warrant further investigation.

Some genes have lower prevalence but exhibit moderate to strong effects (Fig. 6c). Upon inspection, some genes identified by BaGPipe are known to confer resistance to other antibiotic classes, likely due to LD within a shared mobile genetic element (MGE). This reflects the potential for cross-resistance or false-positive associations. However, these observations could also indicate mechanisms of resistance that are not yet fully understood, e.g. a regulatory gene. A detailed comparison of gene hits predicted by AMRFinderPlus and those identified by BaGPipe is summarised in Table 1 (see also Supplementary Table 4).

**Table 1:** Gene hits predicted from AMRFinderPlus and identified from bacterial GWAS by BaGPipe for each of the eight tested antibiotics. For each antibiotic, the corresponding class was identified, and AMRFinderPlus was used to screen for known AMR genes within that class in its database. For example, for ciprofloxacin (cip), a quinolone, genes like *gyrA*, *parC*, and *parE* were predicted from the sequences. There are some gene hits identified by BaGPipe that are not predicted from the known database; these hits are manifested as identifiers extracted from GFF files during annotation. Only the most common genes are shown here, and the full comparison table is Supplementary Table 4. BaGPipe‑only gene functions (where annotated) and concise literature notes on any plausible links to resistance mechanisms are provided in Supplementary Table 7

Antibiotic	Class	Known AMR gene(s) predicted from AMRFinderPlus	Gene hit(s) identified from BaGPipe
cip	Quinolone	*gyrA*	*gyrA*
cip	Quinolone	*parC*	*parC*
cip	Quinolone	*parE*	Not found
cli	Lincosamide	*ermA*	ermA*
cli	Lincosamide	*ermC, lnu(A)*	Not found
ery	Macrolide	*ermA*	*ermA*
ery	Macrolide	*parC*	parC*
ery	Macrolide	*gyrA*	gyrA*
ery	Macrolide	*ermC, mph(C), msr(A)*	Not found
ery	Macrolide	Not found	*GKPKGGHK_02596*
fus	Fusidic acid	*fusA, fusB, fusC*	*fus*
fus	Fusidic acid	*parC*	parC*
fus	Fusidic acid	Not found	*LFPMDBMC_02247, LFPMDBMC_02248, SA0059*
gen	Aminoglycoside	*aadD1*	aadD*
gen	Aminoglycoside	*qacA*	qacA*
gen	Aminoglycoside	*aac(6’)-le/aph(2″)-Ia, ant(6)-Ia, ant(9)-Ia, aph(3’)-IIIa*	Not found
mup	Mupirocin	*ileS*	ileS_2, ileS*
mup	Mupirocin	*parC*	parC*
mup	Mupirocin	*fusA, fusB, fusC*	fus*
mup	Mupirocin	*aadD1*	aadD*
mup	Mupirocin	*mupA*	Not found
oxa	Beta-lactam	*mecR1*	mecR1*
oxa	Beta-lactam	*blaZ, blaI, blaPC1, blaR1, mecA, mecI*	Not found
oxa	Beta-lactam	Not found	*fmtC, fruA*
tet	Tetracycline	*tet(K)*	*tet(K)*
tet	Tetracycline	*fusA, fusB, fusC*	fus*
tet	Tetracycline	*rpoB*	rpoB*
tet	Tetracycline	*tet(M), tet(38), tet(L)*	Not found
tet	Tetracycline	Not found	*cds − M013TW_04140, cds − M013TW_04125*

^*^Not significant gene hit. cip, ciprofloxacin; cli, clindamycin; ery, erythromycin; fus, fusidic acid; gen, gentamicin; mup, mupirocin; oxa, oxacillin; tet, tetracycline

The counts of resistant and susceptible phenotypes for all eight antibiotics, along with Manhattan plots and gene hit plots produced from BaGPipe, are provided in the Supplementary Materials and Zenodo (10.5281/zenodo.14947249).

### Benchmarking BaGPipe against other GWAS tools

To evaluate BaGPipe's computational performance and analytical capabilities, we benchmarked it over the *S. pneumoniae* dataset against two state-of-the-art bacterial GWAS tools: AURORA, a recent machine learning-based method [34], and microGWAS, the latest Snakemake pipeline for bacterial genome-wide association studies [28]. Table 2 summarises the key differences between these approaches across multiple dimensions.

**Table 2:** Comparison of bacterial GWAS tools

Feature	BaGPipe	AURORA	microGWAS
Implementation	Nextflow pipeline	R package	Snakemake pipeline
Statistical Method	Linear Mixed Models (pyseer)	Machine learning: Random Forest, AdaBoost, Logistic Regression	Linear Mixed Models (pyseer)
Genotype Inputs	K-mers/Unitigs, Gene presence/absence, or Variants	Gene presence/absence, or k-mers	Unitigs, Gene presence/absence, Variants, Gene cluster k-mers, or Whole-genome combined (machine learning) unitigs
Required Input Files	Assemblies, Phenotypes	Annotated Genomes, Panaroo/Roary gene presence/absence matrix, phylogeny	Assemblies, Phenotypes, Annotations (GFF)
Annotation	Automatic (Bakta/Prokka), but user can skip this by providing GFF files	External requirement (Panaroo/Roary input)	External requirement (GFF input)
Population Structure	Linear mixed models with kinship matrix (based on phylogenetic correction)	Phylogenetic correction and auto/allopatric strain identification	Linear mixed models with unitig-based kinship
Visualisation	Automatic (Q-Q, Manhattan, Gene-hit plots)	Externally manual	Automatic (Q-Q, Manhattan, Functional enrichment plots)
AMR Integration	Built-in optional module (AMRFinderPlus)	None	Built-in optional module (abritamr)
Screening Significant Genes on External Dataset	Built-in optional module	None	None
Containerisation	Docker/Singularity	None	Conda environments
Level of Automation	End-to-end	GWAS only	Near end-to-end (requires pre-annotation)

After mapping AURORA features to gene symbols via the Panaroo Roary file and restricting to named genes, AURORA yielded 2,801 distinct genes (Supplementary Table 8) and BaGPipe produced 20 genes. The intersection comprised 10 genes, corresponding to a Jaccard index of 0.0036. Top-K overlap was 0 at all K values from 10 to 100, reflecting the fact that the very top of each list is dominated by method-specific features. Spearman rank correlation across the overlapping set was ρ = 0.1405 (p = 0.6986), indicating weak pairwise association (Supplementary Table 9; Supplementary Fig. 10). Despite the modest global overlap, the three canonical β-lactam targets were detected by both methods: *pbp2x*, *pbp2b*, and *pbp1a*. In the BaGPipe results these genes had hits of 178, 74, and 16 respectively; in AURORA they achieved R-class F1 ≈ 0.667 for all three (Supplementary Table 9). Additional overlaps included *mraY*, *recR*, *gpsB*, *rsmH*, *fabG*, *ftsL*, and *malP*, consistent with broader cell-wall and replication/repair associations in the cohort.

**Fig. 7:**
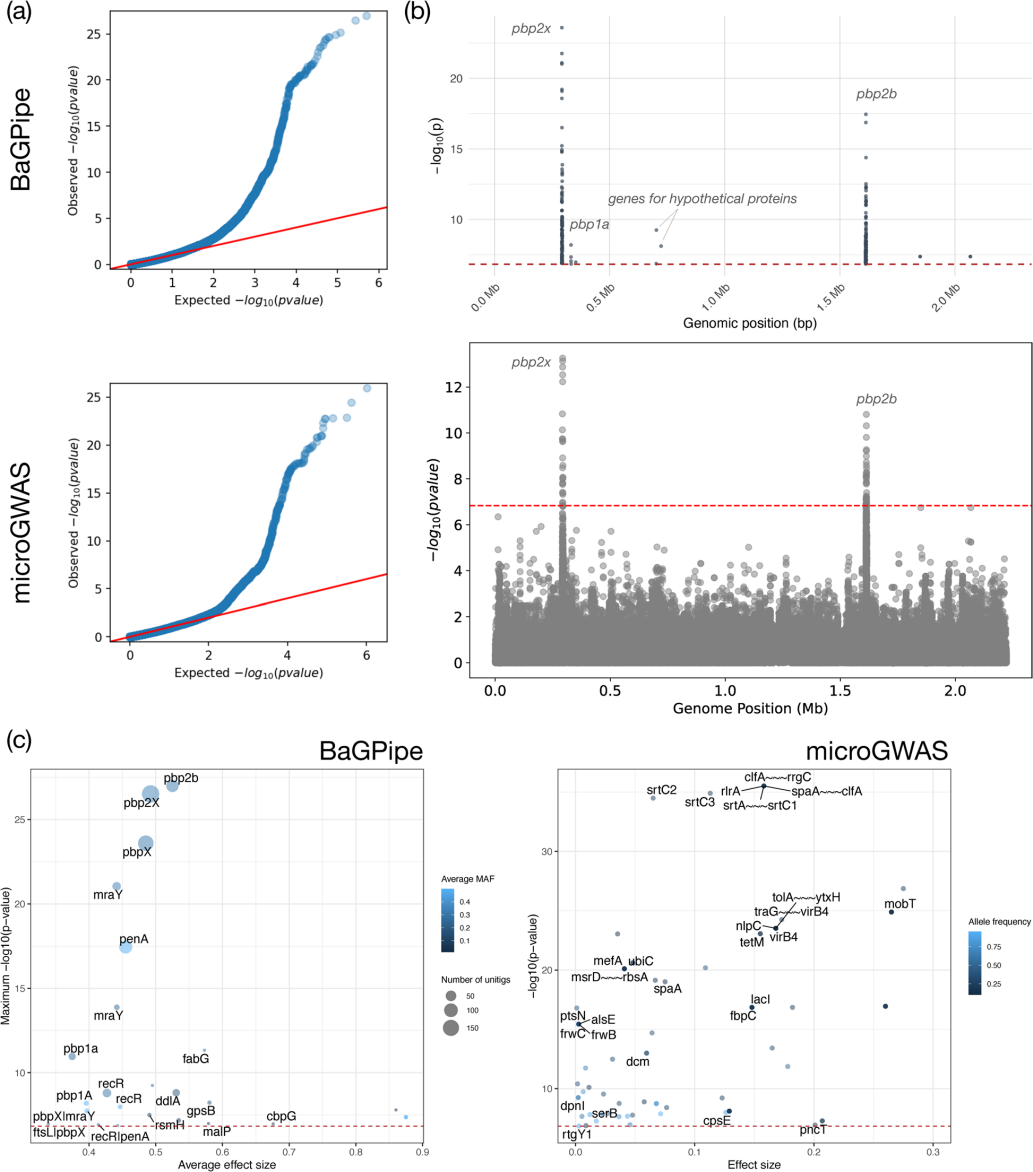
Comparison of the results from BaGPipe and from microGWAS on the *S. pneumoniae* dataset. **a** Q-Q plots, showing that observed − log_10_(*p*-values) are not inflated at low − log_10_(*p*-values) and there is an absence of any poorly controlled confounding population structure (they would appear as big “steps” deviating from the diagonal line). The position of the points being above the null hypothesis (the diagonal line) indicates significant k-mers/unitigs associated with penicillin resistance. **b** Manhattan plots. In both plots, the strongest peak represents the locus coding for the putative penicillin binding protein 2x (*pbp2x* or *pbpX*); the second strongest peak represents *pbp2b* (or *penA*) gene. The red dashed lines are the *p*-value thresholds used by the pipelines to determine an association significant. **c** Gene-hit plots. The x-axis represents the average effect size (or beta), while the y-axis shows the − log_10_(*p*-value), highlighting the statistical significance of gene associations. The size of the circular dots on the BaGPipe’s plot indicates the number of unitigs involved in each association, providing insight into the genomic support for each hit. This information was not obtained from a default microGWAS run, hence lacking on the right. The colour scheme, ranging from lighter to darker shades of blue, illustrates the average MAF for BaGPipe (or allele frequency for microGWAS), with darker shades indicating a lower MAF. The red dashed lines are the *p*-value thresholds used by the pipelines to determine an association significant. Genes which names are only strain-specific locus tags are not labelled on the plots

In comparison, microGWAS yielded 1,407 distinct genes, whereas the intersection with BaGPipe’s results comprised 2 genes, corresponding to a Jaccard index of 0.0014 (Supplementary Table 11). The two overlapping genes were *pbp2x* and *fabG*, however, it was clear that microGWAS also picked up *pbp2b* from the Manhattan plot (Fig. 7), but the association might be lost in the downstream gene mapping. In BaGPipe, *pbp2x* had the highest rank (hits = 178; rank = 1) whereas *fabG* was mid‑list (rank = 14), but both appeared deep in the microGWAS ranking (microGWAS ranks = 678 for *pbp2x* and 947 for *fabG*). Interestingly, the third highest signal identified from BaGPipe is the *pbp1a* gene, which was not identified by microGWAS (Fig. 7).

Taken together, our comparative analysis on the *S. pneumoniae* penicillin resistance dataset revealed that while the three methods show relatively low global feature overlap (reflecting their distinct statistical frameworks and filtering strategies, and differences in significant gene annotation mapping), they consistently identify the most significant well-established biological associations, the canonical β-lactam resistance determinants. AURORA's machine learning framework excels at predictive accuracy and capturing non-linear associations; microGWAS offers flexibility through six complementary statistical approaches; and BaGPipe prioritises interpretability and automation for gene-level biological discovery.

From a computational perspective, the successful main microGWAS run completed with approximately 44 CPU-hours with peak memory usage of 7.2 GB on this moderately-sized dataset (616 strains). Resource requirements are slightly higher than BaGPipe (without genome annotation). The analysis generated 3.0 GB of output data with 8.0 GB for intermediate Snakemake files. Both tools demonstrated resource profiles typical of multi-step bacterial genomics pipelines on HPC environments. To get the workflow running for new users, both pipelines required some debugging attempts, though BaGPipe's modular Nextflow design with extensive caching drastically reduces re-computation costs for parameter exploration, as does microGWAS’s Snakemake rule-based dependency tracking.

To further validate BaGPipe's performance, we tested it on a published *Escherichia coli* virulence dataset [44] originally reported by the microGWAS authors [28]. BaGPipe successfully identified *irp1*, *irp2*, and *ybtU* as significantly associated with the phenotype. These genes comprise the yersiniabactin biosynthesis cluster (located at approximately 2 Mb), a well-characterized pathogenicity island associated with enhanced virulence in extraintestinal pathogenic *E. coli*. BaGPipe also detected significant associations at location 1.15 Mb, corresponding to the high-pathogenicity island (HPI), in agreement with microGWAS findings. These results demonstrate BaGPipe's ability to recapitulate known biology and detect established genetic determinants across different bacterial species and phenotypes (Results included in Supplementary Materials 12–15).

## Discussion

BaGPipe addresses the challenges associated with analysing large-scale bacterial datasets by integrating a cutting-edge workflow management tool, Nextflow [29], along with a modular design that allows researchers to efficiently conduct end-to-end analyses. This pipeline offers an accessible solution to a major bottleneck in bacterial genomics: performing reproducible and efficient GWAS on large datasets with diverse computational requirements.

Validation of BaGPipe through replication of a published *S. pneumoniae* GWAS study [30–32] demonstrated optimised computational performance. Further application to a *S. aureus* dataset illustrated its versatility across different microbial contexts, with cross-validation using AMRFinderPlus [40] identifying both established AMR genes and previously unreported genetic elements. While some of these loci are not confirmed resistance genes, they represent promising leads for further experimental investigation, as these associations do not necessarily indicate false positives. For instance, mutations in *rpoB*, initially linked to rifaximin resistance, were recently shown to confer daptomycin resistance in *Enterococcus faecium* [19]. These findings highlight both BaGPipe’s sensitivity and robustness in hypothesis generation for discovering unexplored genetic mechanisms.

Our comprehensive benchmarking of BaGPipe against two state-of-the-art bacterial GWAS tools, AURORA and microGWAS, shows that BaGPipe offers a distinctive, complementary approach to bacterial association analysis. Rather than a single optimal method, the field benefits from multiple specialised tools suited to different research aims, computational constraints, and levels of user experience. BaGPipe’s strengths lie in its modularity, flexibility, and automated resource management, which streamline analyses without manual adjustments. When isolating the association step, BaGPipe finished in under eight minutes of CPU time while producing all standard outputs, whereas the machine learning based AURORA required ~ 58 min of wall-time and additional downstream mapping from pangenome clusters to gene symbols. Biologically, BaGPipe prioritised the canonical penicillin-resistance determinants in *S. pneumoniae* (*pbp2x*, *pbp2b*, *pbp1a*), consistent with established literature and the pyseer tutorial, enabling rapid interpretation. Although AURORA detected these loci, they were not ranked among the top features, and interpretation was delayed by the need for manual feature annotation. microGWAS agreed with BaGPipe in identifying the top penicillin-resistance genes (*pbp2x* and *pbp2b*), but did not recover the weaker *pbp1a* signal detected by BaGPipe. microGWAS provides greater flexibility through six complementary statistical approaches and may deliver additional insights if allocated more resources. On this moderately-sized dataset (616 strains), the main microGWAS run completed in ~ 44 CPU-hours with a peak memory usage of 7.2 GB; resource requirements were slightly higher than BaGPipe (without genome annotation). Excluding genome annotation to match the microGWAS setup, BaGPipe required ~ 19 CPU-hours on the same dataset. Uniquely, BaGPipe integrates genome annotation, AMR prediction, GWAS analysis, and an external validation workflow within a single, reproducible framework that requires only raw assemblies and phenotype data. This end-to-end automation substantially reduces the bioinformatics burden, making BaGPipe particularly valuable for research groups without dedicated computational expertise.

From a computational perspective, BaGPipe's implementation in Nextflow provides significant practical advantages. Nextflow is one of the most widely adopted workflow managers in genomics, ensuring immediate familiarity to the bioinformatics community and access to extensive documentation, training resources, and community support. The highly modular architecture of BaGPipe enables researchers to customize analyses: users can provide their own phylogenetic trees (critical for population structure correction), swap tools to leverage newer methods, extend pipelines, or integrate BaGPipe with other established workflows in a standardised manner. This modularity makes BaGPipe a robust long-term choice that can evolve alongside methodological advances, new input formats, and pathogen-specific requirements, essential for a field advancing as rapidly as bacterial genomics.

BaGPipe is actively maintained by the Pathogen Informatics team at the Wellcome Sanger Institute, ensuring regular updates, responsive issue tracking, and integration of user feedback from the research community. This institutional commitment to sustainability differentiates BaGPipe from many academic pipelines that lack long-term support structures. The combination of comprehensive functionality, computational efficiency, active maintenance, and integration with the broader Nextflow ecosystem positions BaGPipe as an accessible and sustainable tool for advancing bacterial genetic research, with the potential to become indispensable for researchers seeking an integrated solution for microbial association studies. With continued improvements and community-driven development, BaGPipe has the potential to foster new discoveries and deepen our understanding of bacterial genetics and pathogenesis.

## Conclusions

BaGPipe is a powerful, flexible, and efficient pipeline for conducting bacterial GWAS with the well-established Pyseer framework. We show it is capable of reliably handling complex datasets, reducing redundancy, and replicating or uncovering both known and novel genetic associations related to antibiotic resistance. BaGPipe lowers the barriers to performing bacterial GWAS and offers a comprehensive, reproducible framework, vital to better understand the genetic basis of AMR and bacterial traits.

## Supplementary Information

Supplementary Material 1. Supplementary Tables 1-5, 7-9, 11, 13.

Supplementary Material 2. Supplementary 6, 10, 12, 14-16.

## Data Availability

BaGPipe is freely available at [https://github.com/sanger-pathogens/BaGPipe] (https://github.com/sanger-pathogens/BaGPipe). The *Streptococcus pneumoniae* input dataset is available from the Pyseer tutorial ([https://pyseer.readthedocs.io/en/master/tutorial.html#] (https://pyseer.readthedocs.io/en/master/tutorial.html). The *Staphylococcus aureus* sequencing assemblies can be sourced from their ERS accession numbers provided in supplementary data. The reference assemblies, listed in the supplementary, can be sourced from NCBI. Supplementary data are available at [https://doi.org/10.5281/zenodo.14947249] (https://doi.org/10.5281/zenodo.14947249).

## Abbreviations

AMR: Antimicrobial Resistance
GWAS: Genome-wide Association Study
HPC: High-performance Computing
HPI: High-pathogenicity Island
Indel: Insertions and Deletions
LD: Linkage Disequilibrium
LSF: Load Sharing Facility
MAF: Minimum Allele Frequency
MDS: Multidimensional Scaling
MGE: Mobile Genetic Element
MRSA: Methicillin-Resistant *Staphylococcus aureus*
Q-Q plot: Quantile–Quantile plot
RAM: Remote-Access Memory
SNP: Single Nucleotide Polymorphism
VCF: Variant Calling Format
