# Supplementary material for: BaGPipe: an automated, reproducible, and flexible pipeline for bacterial genome-wide association studies
Source: BMC Microbiol. 2026 Mar 27;26:441. doi: 10.1186/s12866-026-04909-9 (PMC13147680; doi:10.1186/s12866-026-04909-9)
Supplement: Supplementary file 2 — Supplementary Material 2. Supplementary 6, 10, 12, 14-16. [file 12866_2026_4909_MOESM2_ESM.pdf]

Supplementary 1 (Table): Current prominent methods in overcoming bacterial GWAS challenges. Most tools demand additional software and expertise from the user to execute complex analyses. BH adjustments, Benjamin-Hochberg adjustments; FaST-LMM, Factored Spectrally Transformed Linear Mixed Models; MDS, Multidimensional Scaling; PCA, Principal Component Analysis. Attached separately as an Excel file.

Supplementary 2 (Table): Metadata of all 520 *Staphylococcus aureus* assemblies including their ERS accession numbers. Attached separately as an Excel file.

Supplementary 3 (Table): Data for Top Five Species Plot for MultiQC report produced from BacQC (Kraken2 and Bracken) on the *Staphylococcus aureus* assemblies. Attached separately as an Excel file.

Supplementary 4 (Table): Summary of gene-level calls identified exclusively by BaGPipe, exclusively by AMRFinderPlus, or jointly by both, from the *Staphylococcus aureus* study. Columns denote independent runs, each corresponding to a different target phenotype. Attached separately as an CSV file.

Supplementary 5 (Table): Number of resistant and susceptible *Staphylococcus aureus* isolates for each tested antibiotic. Attached separately as an Excel file.

Supplementary 6: Reference genome assemblies used in the implementation of BaGPipe in the analysis with the *Staphylococcus aureus* dataset. Assembly accession number are shown here and these can be searched on the NCBI Assembly database.

1. GCA_000009645.1_ASM964v1
2. GCA_000237125.3_ASM23712v3
3. GCA_000953255.1_Staphylococcus_aureus_Sa_ILRI_217
4. GCA_000011265.1_ASM1126v1
5. GCA_000017085.1_ASM1708v1
6. GCA_001611405.1_ASM161140v1
7. GCA_000009585.1_ASM958v1
8. GCA_000412775.1_ASM41277v1

Supplementary 7 (Table): BaGPipe-only genes in the *S. aureus* study compared to AMRFinderPlus predictions. Functional assignments were compiled from primary literature and databases. The ‘antibiotic’ column indicates the phenotype context; uncurated products are marked hypothetical. Attached separately as an Excel file.

Supplementary 8 (Table): AURORA penicillin-resistant-associated top hits in the *S. pneumoniae* dataset. Each row lists a pangenome feature (“Variant”) identified by AURORA GWAS when modelling the resistant (R) class. Columns report R std_residual, R precision, R recall, and the R F1 score used for ranking (descending). “Variant” names are either Panaroo/Roary cluster IDs (e.g., group_####) or mapped gene symbols; multiple symbols joined by “~~~” indicate co-annotations within the same cluster/feature. “NA” in R std_residual denotes that AURORA did not compute a residual for that feature in this run. Metrics follow AURORA’s output conventions for per-class evaluation. Attached separately as an CSV file.

Supplementary 9 (Table): Gene-level overlap between AURORA and BaGPipe (*S. pneumoniae*). Genes present in both analyses are listed with their per-method scores and within-method ranks. AURORA per-gene evidence summarised as the maximum R-class F1 across mapped pangenome features for that gene (higher is better) and its rank within the AURORA gene list (1 = best). Values derive from the AURORA GWAS output after mapping Panaroo/Roary clusters to gene names. BaGPipe gene-level evidence summarised by the gene_hits’ ‘hits’ metric (or the highest available gene-level score) and its rank within the BaGPipe list (1 = best). Attached separately as an CSV file.

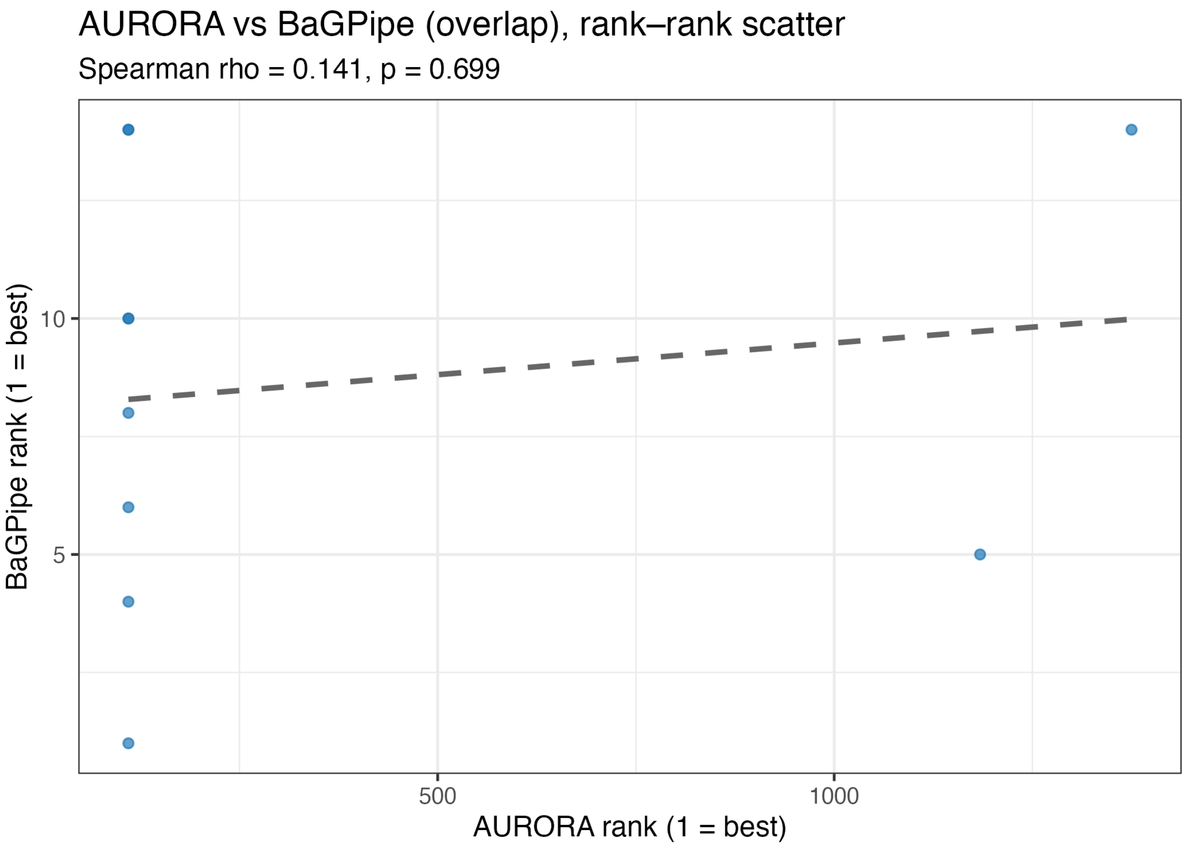

Supplementary 10: AURORA-BaGPipe rank-rank concordance (*S. pneumoniae*). Scatterplot of genes shared between AURORA and BaGPipe (n = 10). The x-axis shows AURORA rank (1 = best), computed from the maximum R-class F1 per gene after mapping Panaroo/Roary clusters to gene names; the y-axis shows BaGPipe rank (1 = best) from the gene_hits' "hits" metric. The dashed line is an ordinary least-squares fit. Spearman's $\rho = 0.141$, $p = 0.699$, indicating weak monotonic concordance across overlapping genes. Canonical β-lactam loci (*pbp2x*, *pbp2b*, *pbp1a*) are included among the points.

Supplementary 11 (Table): microGWAS gene-presence/absence associations in *Streptococcus pneumoniae*. Each row lists a pan-genome feature tested by microGWAS. Reported columns are: variant (feature identifier), af (allele frequency of the feature across isolates), filter-pvalue (pre-test filter statistic reported by the pipeline), lrt-pvalue(likelihood-ratio test *p*-value from the logistic association for presence/absence), beta (effect size), beta-std-err (standard error of beta), variant_h2 (per-variant heritability estimate), and notes (quality flags; entries marked bad-chisq indicate model-fit warnings and should be interpreted with caution). Attached separately as an TSV file.

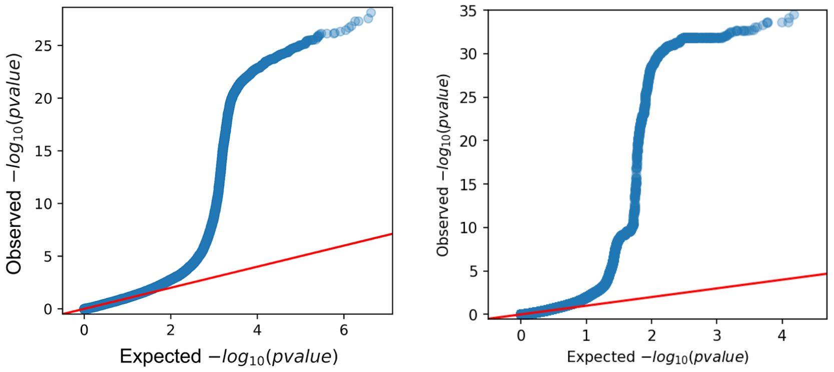

Supplementary 12: Comparison of Q-Q plots from microGWAS (left) and BaGPipe (right) on the *E. coli* dataset. The Q-Q plots show that observed $-\log_{10}$(p-values) are not inflated at low $-\log_{10}$(p-values) and there is an absence of any poorly controlled confounding population structure (they would appear as big "steps" deviating from the diagonal line). The position of the points being above the null hypothesis (the diagonal line) indicates significant k-mers/unitigs associated with virulence. The Q-Q plot produced from microGWAS was sourced from the microGWAS paper (Burgaya et al., 2024).

Supplementary 13 (Table): BaGPipe gene-level hit summary for the *E. coli* dataset. Columns are hits (number of associated features assigned to the gene under the study's significance/filtering settings), maxp (maximum $-\log_{10} p$ across features mapped to the gene), avg_af (mean allele frequency across mapped features), avg_maf (mean minor-allele frequency), and avg_beta (mean effect size across mapped features). Attached separately as an TSV file.

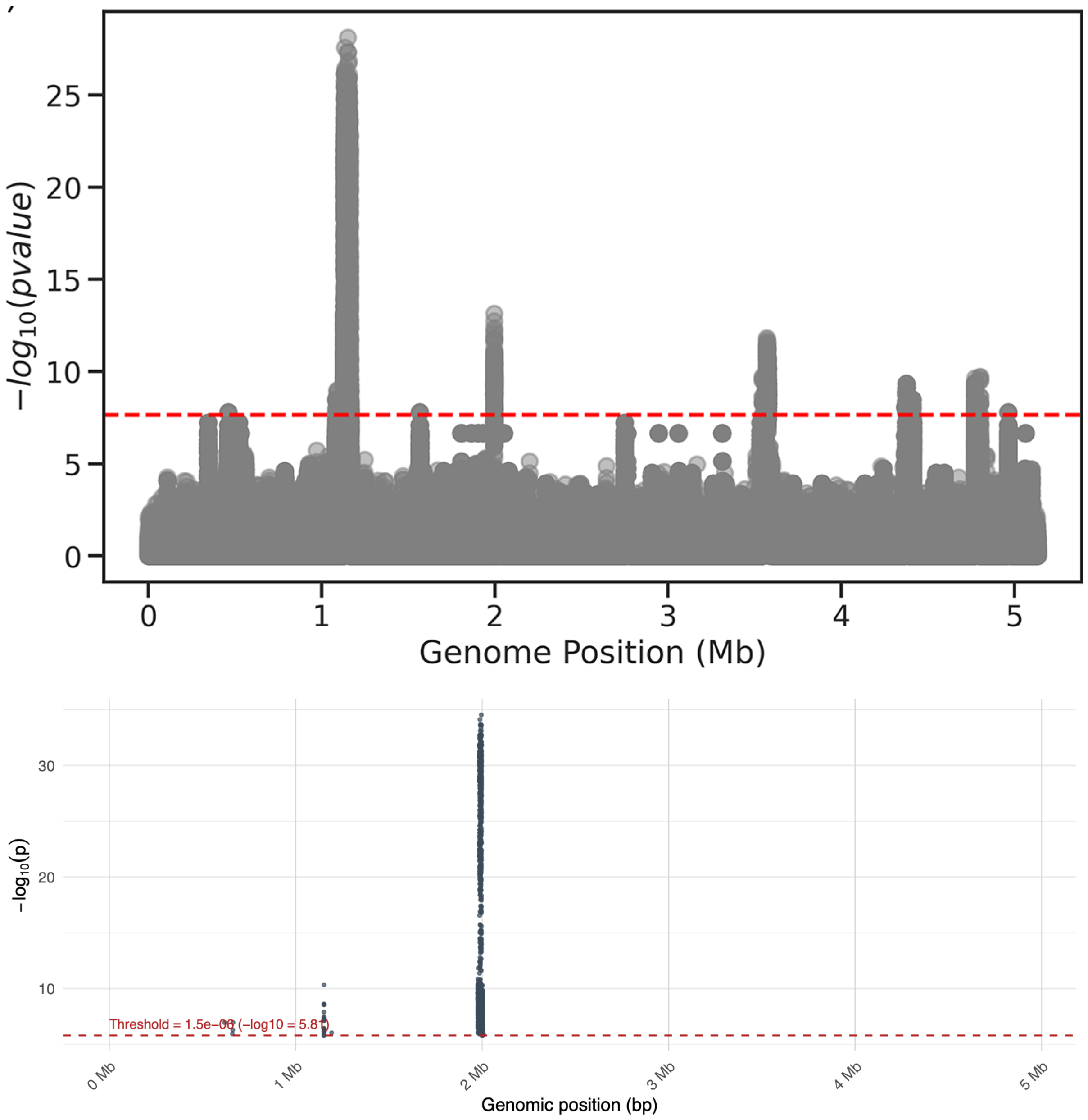

Supplementary 14: Comparison of the Manhattan plots from microGWAS (top) and from BaGPipe (bottom) on the *E. coli* dataset. The strongest peak (2Mb) represents the locus coding for the yersiniabactin biosynthesis cluster (comprising *irp1*, *irp2*, and *ybtU*). BaGPipe also detected significant associations at location 1.15 Mb, corresponding to the high-pathogenicity island (HPI), in agreement with microGWAS findings. The Manhattan plot produced from microGWAS was sourced from the microGWAS paper (Burgaya et al., 2024).

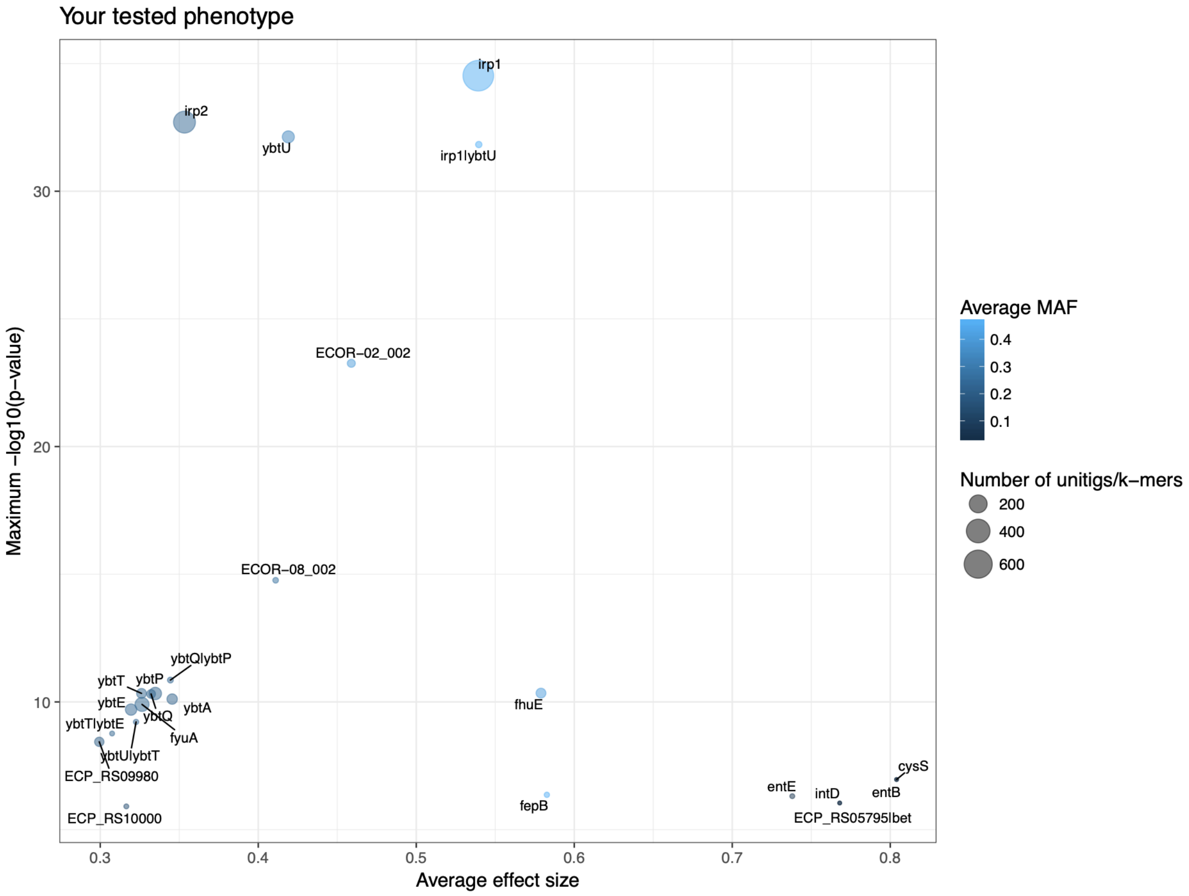

Supplementary 15: Gene-Hit plots from BaGPipe on the *E. coli* dataset. The x-axis represents the average effect size, while the y-axis shows the $-\log_{10}(p\text{-value})$, highlighting the statistical significance of gene associations. The size of the circular dots on the BaGPipe's plot indicates the number of unitigs involved in each association, providing insight into the genomic support for each hit.

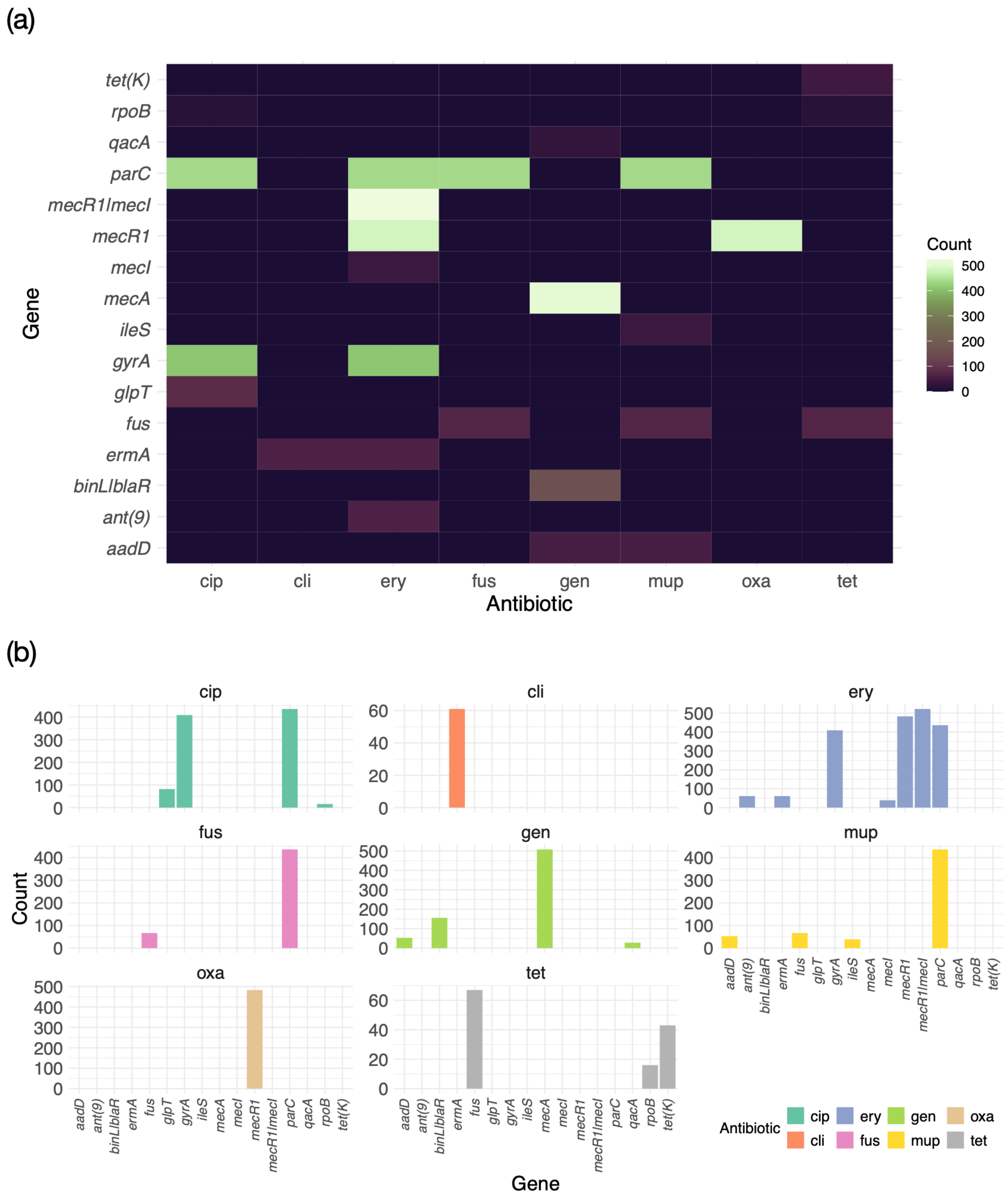

Supplementary 16: Frequency of matched gene hits from BaGPipe GWAS analysis and AMRFinderPlus prediction in the eight tested antibiotics for the *S. aureus* dataset. (a) Heatmap and (b) faceted bar plot of the frequency of matched genes across the eight tested antibiotics. The bars represent the counts of genomes in which gene hits identified by BaGPipe and predictions by AMRFinderPlus coincide, indicating the prevalence of known AMR genes within the tested strains. BaGPipe successfully identified known AMR genes in every tested antibiotic. cip, ciprofloxacin; cli, clindamycin; ery, erythromycin; fus, fusidic acid; gen, gentamicin; mup, mupirocin; oxa, oxacillin; tet, tetracycline.
